# Unique Mānuka Factor (UMF)-dependent antimicrobial activity of Mānuka honey against respiratory pathogens cannot be explained by sugar and methylglyoxal alone

**DOI:** 10.1099/mic.0.001746

**Published:** 2026-08-11

**Authors:** Gemma J. Allcott, Jackie Evans, Troy L. Merry, Jonathan A. G. Cox

**Affiliations:** 1 School of Biosciences, Aston University, Birmingham, UK; 2 Comvita Ltd, Te Puke, New Zealand; 3 Faculty of Medical and Health Sciences, University of Auckland, Auckland, New Zealand

**Keywords:** antimicrobial resistance, infection, Mānuka honey, pathogens, respiratory

## Abstract

Respiratory bacterial infections remain a major cause of morbidity and mortality worldwide, and their treatment is increasingly complicated by antimicrobial resistance. Mānuka honey has attracted interest as a potential alternative antimicrobial agent, although the extent to which antibacterial activity varies with Unique Mānuka Factor (UMF™) grade and the relative contribution of methylglyoxal (MGO) remain unclear. In this study, the antimicrobial activity of five UMF™ grades of Mānuka honey (5+, 10+, 12+, 15+ and 20+) was evaluated against clinically relevant respiratory pathogens: methicillin-susceptible *Staphylococcus aureus* (MSSA), methicillin-resistant *S. aureus* (MRSA), *Klebsiella pneumoniae* and *Pseudomonas aeruginosa*. MICs were determined using broth microdilution assays. To assess the contribution of osmotic stress, bacterial growth responses were compared with a sugar solution matched to the carbohydrate composition of honey. In addition, MGO-matched control solutions were prepared to examine the contribution of MGO to antimicrobial activity. All Mānuka honey samples inhibited bacterial growth, and MIC values decreased with increasing UMF™ grade. MSSA and MRSA were the most susceptible organisms, whereas higher concentrations were required to inhibit the Gram-negative species. Mānuka honey consistently inhibited bacterial growth more strongly than the matched sugar control, indicating that osmotic effects alone do not explain its antimicrobial activity. Although MGO-matched solutions exhibited antibacterial activity, they were generally less potent than the corresponding whole honey samples. These findings demonstrate that Mānuka honey exhibits UMF-dependent antimicrobial activity against respiratory pathogens and suggest that its antibacterial effects arise from the combined action of MGO and additional components within the honey matrix

**Impact Statement**

Antimicrobial resistance is making bacterial respiratory infections increasingly difficult to treat, highlighting the need to explore alternative or complementary antimicrobial strategies. Mānuka honey has attracted considerable interest because of its antibacterial activity, but most studies have focused on wound pathogens rather than respiratory organisms. In addition, the relative contribution of its chemical components to antimicrobial activity remains incompletely understood. In this study, we evaluated the activity of multiple Unique Mānuka Factor (UMF™) grades of Mānuka honey against clinically relevant respiratory pathogens, including *Staphylococcus aureus*, *Klebsiella pneumoniae* and *Pseudomonas aeruginosa*. By comparing honey with sugar-matched and methylglyoxal-matched control solutions, we show that increasing UMF grade is associated with stronger antibacterial activity and that this activity cannot be explained by sugar content or methylglyoxal alone. These findings contribute to understanding the multifactorial antimicrobial properties of Mānuka honey and support further investigation of its potential applications in respiratory infection research.

## Data Summary

All data generated or analysed during this study are included within this article.

The authors confirm all supporting data, code and protocols have been provided within the article or through supplementary data files.

## Introduction

Respiratory bacterial infections remain a major cause of morbidity and mortality worldwide, and their clinical management is increasingly complicated by antimicrobial resistance (AMR). Pathogens such as *Staphylococcus aureus*, *Klebsiella pneumoniae* and *Pseudomonas aeruginosa* are important causes of respiratory disease in both community and healthcare settings, where multidrug-resistant phenotypes can substantially restrict treatment options and lead to poorer clinical outcomes [1, 2]. The continued rise of AMR, coupled with the limited pace of antibiotic discovery, has intensified interest in alternative or adjunct antimicrobial strategies [3, 4].

Mānuka honey, a monofloral honey derived from the nectar of *Leptospermum scoparium* in New Zealand, has attracted considerable attention because of its broad-spectrum antimicrobial activity [5, 6]. Unlike other honeys, the antibacterial activity of Mānuka honey is largely attributed to non-peroxide mechanisms [6, 7]. Commercial Mānuka honey is commonly classified using the Unique Mānuka Factor (UMF™) grading system, which reflects authenticity and the presence of key marker compounds. The UMF™ grading system is independently certified and incorporates measurements of methylglyoxal (MGO), leptosperin and dihydroxyacetone, alongside tests confirming the absence of adulteration. UMF™ grades typically range from 5+ to 20+ or higher, with increasing grades generally associated with higher concentrations of MGO. However, whether increasing UMF™ grade consistently translates into greater antibacterial activity remains unclear [7, 8].

Although the antimicrobial properties of Mānuka honey are well documented, much of the available literature has focused on wound-associated organisms, particularly *S. aureus* [9–12]. In contrast, comparatively little work has systematically examined its activity against clinically relevant respiratory pathogens. In addition, because honey is a highly concentrated sugar solution, osmotic stress is recognized as an important contributor to its antimicrobial activity [13, 14]. However, the sugar composition of Mānuka honey is broadly comparable to that of other honeys, yet Mānuka frequently exhibits greater antibacterial activity, suggesting that additional bioactive components contribute to its antimicrobial effects [15, 16]. Distinguishing the contribution of sugar from other antimicrobial components therefore represents an important step in understanding the biological activity of Mānuka honey.

MGO, a highly reactive dicarbonyl compound capable of modifying proteins and nucleic acids through glycation, has been identified as a defining chemical component of Mānuka honey [17]. MGO has frequently been proposed as the principal driver of the non-peroxide antimicrobial activity associated with Mānuka honey [18, 19]. However, this interpretation appears to be somewhat simplified. Several studies have reported inconsistent relationships between MGO concentration, UMF™ grade and antibacterial activity, indicating that antimicrobial effects do not consistently correlate with MGO levels in Mānuka honey [7, 20]. These discrepancies highlight ongoing uncertainty regarding the extent to which MGO alone accounts for the non-peroxide antibacterial activity of Mānuka honey. Indeed, recent research suggests that other compounds in Mānuka honey, such as the phenolic compound 3-phenyllactic acid, potentiate the antibacterial effects of MGO in Mānuka honey [21]. It is therefore important to clarify the relative contribution of MGO to the antimicrobial effects in order to optimize therapeutic formulations which are well characterized.

The aim of this study was to evaluate the antimicrobial activity of five UMF™ grades of Mānuka honey against a panel of clinically relevant respiratory pathogens: methicillin-susceptible *S. aureus* (MSSA), methicillin-resistant *S. aureus* (MRSA), *K. pneumoniae* and *P. aeruginosa*. Antimicrobial activity was assessed using MIC assays, and bacterial growth responses were compared with a matched sugar control to determine whether inhibition could be explained by osmotic effects alone. In addition, MGO-matched control solutions were evaluated to examine the extent to which MGO contributes to the antimicrobial activity of Mānuka honey. Together, these experiments were designed to establish whether Mānuka honey demonstrates UMF-dependent antimicrobial activity against respiratory pathogens and to explore the relative contribution of MGO to this effect.

## Methods

MSSA (MSSA ATCC 29213), MRSA (MRSA N315), *K. pneumoniae* (NCTC 14323) and *P. aeruginosa* (NCTC 13437) were maintained as glycerol stocks at −80 °C. Prior to experimentation, strains were streaked onto Mueller–Hinton agar and incubated at 37 °C for 18–24 h. Single colonies were inoculated into Mueller–Hinton broth and cultured overnight at 37 °C with shaking (180 r.p.m.).

Mānuka honey samples (UMF 5+, 10+, 12+, 15+ and 20+) were provided by Comvita® (New Zealand). Honey was dissolved in sterile distilled water at 37 °C with shaking to ensure complete dissolution and sterilized by filtration through a 0.2 µm membrane filter. Samples were prepared fresh at the beginning of each experiment. Batch-specific compositional data for the Mānuka honey samples are provided in Table S1 (available in the online Supplementary Material).

### Determination of MICs of Mānuka honey

Antimicrobial activity was assessed by broth microdilution in sterile 96-well plates. Overnight cultures of each bacterial species were adjusted to OD_600_=0.1 prior to inoculation using a spectrophotometer. Honey solutions were serially diluted in sterile distilled water to produce final assay concentrations ranging from 0 to 45% (w/v). Each well contained 90 µl test solution and 10 µl of the standardized bacterial suspension prepared in Mueller–Hinton broth (total volume 100 µl). Untreated control wells consisted of 90 µl sterile distilled water and 10 µl of the standardized bacterial suspension. Plates were incubated statically at 37 °C for 20 h, and bacterial growth was quantified by measuring absorbance at 570 nm using a microplate reader. OD_600_ was used for inoculum standardization, whereas OD_570_ was used for endpoint growth measurements based on the instrumentation available. Experiments were performed with four biological replicates per bacterial species, each including three technical replicates. MIC values were defined as the lowest concentration resulting in ≥95% inhibition of bacterial growth relative to the untreated control.

### Sugar control comparison

A sugar control was prepared to approximate the sugar composition of Mānuka honey (82% total sugars), consisting of glucose (32% w/v), fructose (40% w/v) and sucrose (10% w/v). The solution was dissolved in deionized water and sterilized by filtration (0.2 µm). Sugar control solutions were tested using the same broth microdilution assay and dilution range as the honey samples. Experiments were performed with four biological replicates and three technical replicates per concentration. Bacterial growth responses to Mānuka honey and the sugar control were compared using multiple t-tests with Holm–Sidak correction in GraphPad Prism (v10.2.2).

### MGO-matched control solutions

MGO concentrations in each Mānuka honey sample were determined by Comvita New Zealand Ltd. using aqueous extraction followed by derivatization with *o*-phenylenediamine to form a stable quinoxaline derivative, which was subsequently analysed by HPLC-UV detection, as described by Windsor *et al*. [22]. The analytical method employed a calibration range of 0–1,600 mg kg⁻¹ MGO. Quality assurance procedures included the analysis of three levels of quality control material within each analytical batch, consisting of homogeneous honey samples with established MGO concentrations verified through independent analysis by two accredited external laboratories. An internal standard was also included to verify pipetting accuracy.

MGO analyses were performed by an IANZ-accredited laboratory operating under ISO/IEC 17025 quality management systems and subject to routine external auditing. Measurements were conducted as part of standard laboratory workflows without specific knowledge of their subsequent use in this study.

To assess the contribution of MGO to antimicrobial activity, control solutions were prepared by adding exogenous MGO to either distilled water or the sugar control matrix at concentrations equivalent to those measured in each UMF grade. MGO stock solutions (1 g ml⁻¹) were prepared in sterile distilled water and sterilized by filtration (0.2 µm). MGO-matched preparations were serially diluted using the same dilution scheme as the honey samples and tested using the broth microdilution assay described above. Three technical replicates were performed per concentration.

## Results

### Mānuka honey exhibits UMF-dependent antimicrobial activity against respiratory pathogens

**Fig. 1.**
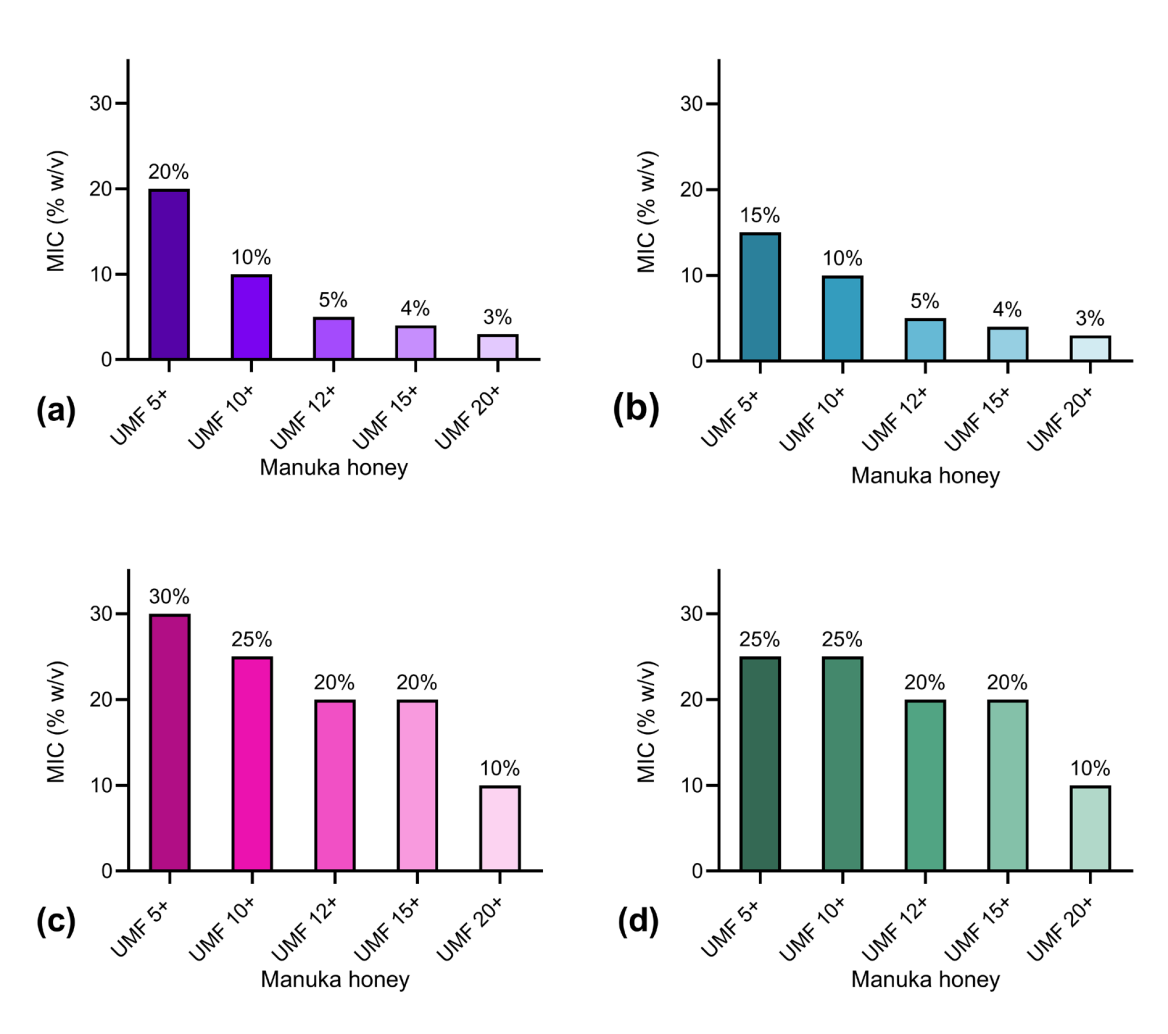
Antimicrobial activity of Mānuka honey against respiratory pathogens. MICs (% w/v) of Mānuka honey with UMF ratings of 5+, 10+, 12+, 15+ and 20+ against (**a**) MSSA, (**b**) MRSA, (**c**) *K. pneumoniae* and (**d**) *P. aeruginosa*. MICs were determined using broth microdilution assays. Experiments were performed using four independent biological replicates, and identical MIC values were obtained in all replicates.

MICs were determined for five UMF grades of Mānuka honey against MSSA, MRSA, *K. pneumoniae* and *P. aeruginosa* (Fig. 1). Antimicrobial activity was observed for all honey samples against all species tested. A clear reduction in MIC values was observed as UMF grade increased, indicating stronger antimicrobial activity at higher UMF levels.

MSSA and MRSA were the most susceptible organisms (Fig. 1a, b), with MIC values decreasing from 20 to 15% (w/v) at UMF 5+ to 3% (w/v) at UMF 20+. In contrast, the Gram-negative species required higher honey concentrations for inhibition (Fig. 1c, d). *K. pneumoniae* and *P. aeruginosa* exhibited MIC ranges of 30–10% (w/v) and 25–10% (w/v), respectively.

### Mānuka honey inhibits bacterial growth more strongly than an equivalent sugar solution

**Fig. 2.**
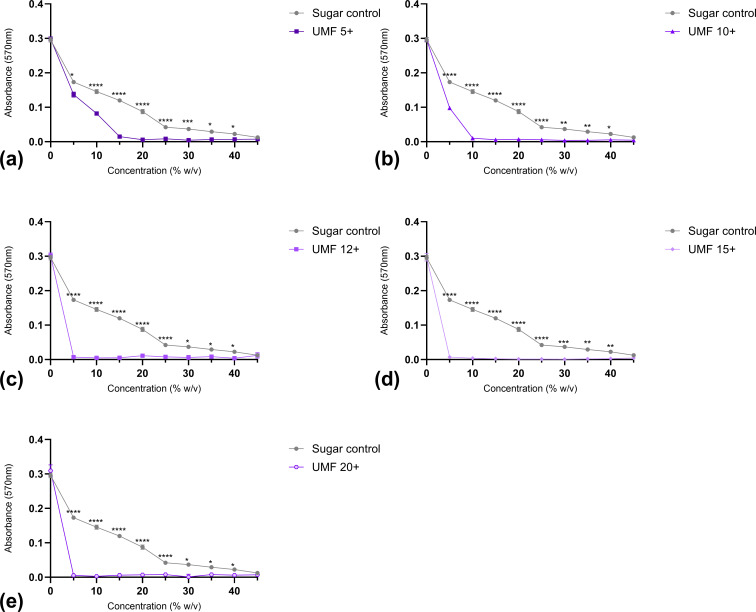
Growth of MSSA in response to increasing concentrations of Mānuka honey. Bacterial growth (absorbance at 570 nm) of MSSA following exposure to increasing concentrations of Mānuka honey with UMF grades of (**a**) 5+, (**b**) 10+, (**c**) 12+, (**d**) 15+ and (**e**) 20+, compared with a sugar control. Data represent four independent biological replicates and are presented as mean±sem. Statistical significance relative to the sugar control was determined using multiple *t*-tests with Holm–Sidak correction (*P*<0.05, *P<0.01, **P<0.005 and ***P<0.001).

**Fig. 3.**
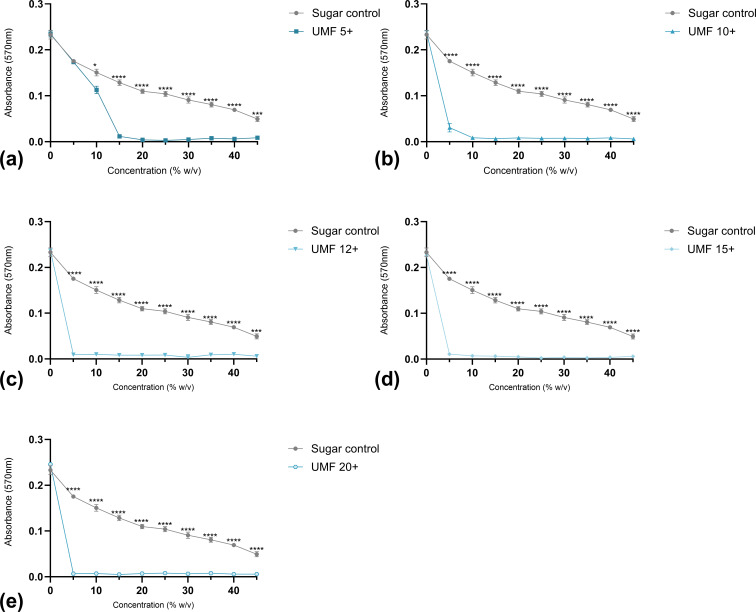
Growth of MRSA in response to increasing concentrations of Mānuka honey. Bacterial growth (absorbance at 570 nm) of MRSA following exposure to increasing concentrations of Mānuka honey with UMF grades of (**a**) 5+, (**b**) 10+, (**c**) 12+, (**d**) 15+ and (**e**) 20+, compared with a sugar control. Data represent four independent biological replicates and are presented as mean±sem. Statistical significance relative to the sugar control was determined using multiple *t*-tests with Holm–Sidak correction (*P*<0.05, *P<0.01, **P<0.005 and ***P<0.001).

**Fig. 4.**
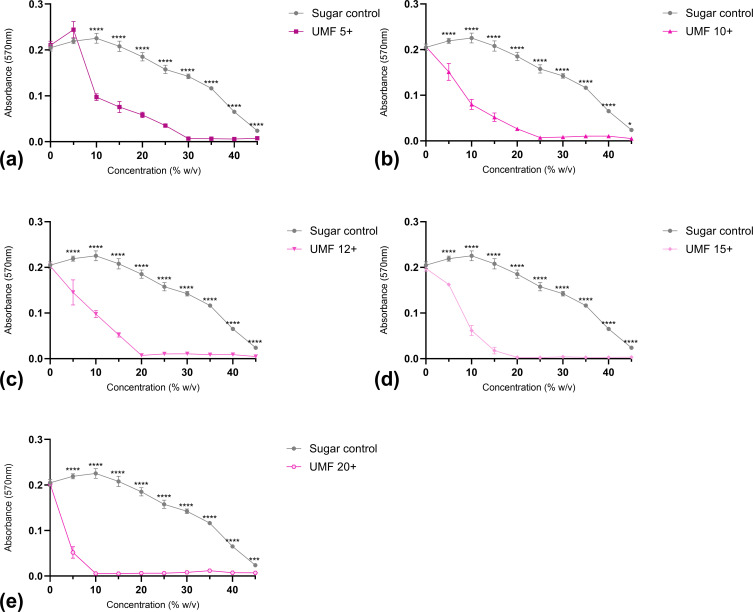
Growth of *K. pneumoniae* in response to increasing concentrations of Mānuka honey. Bacterial growth (absorbance at 570 nm) of *K. pneumoniae* following exposure to increasing concentrations of Mānuka honey with UMF grades of (**a**) 5+, (**b**) 10+, (**c**) 12+, (**d**) 15+ and (**e**) 20+, compared with a sugar control. Data represent four independent biological replicates and are presented as mean±sem. Statistical significance relative to the sugar control was determined using multiple *t*-tests with Holm–Sidak correction (*P*<0.05, *P<0.01, **P<0.005 and ***P<0.001).

**Fig. 5.**
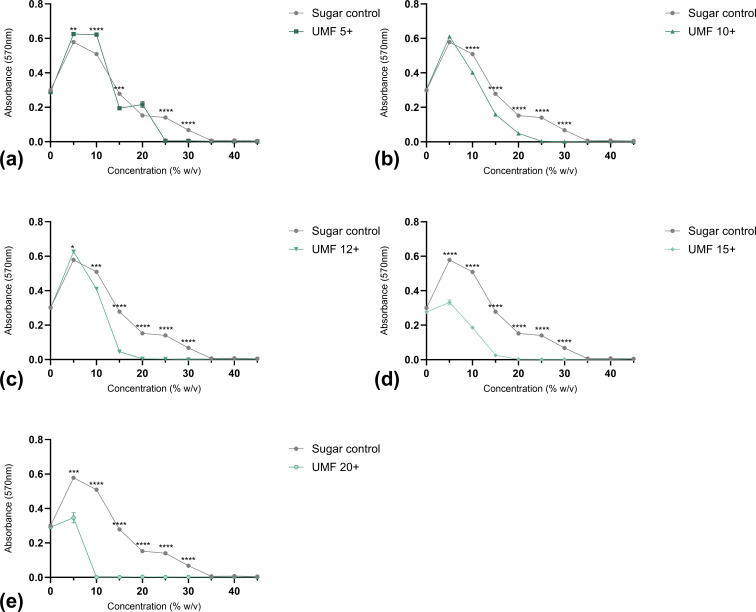
Growth of *P. aeruginosa* in response to increasing concentrations of Mānuka honey. Bacterial growth (absorbance at 570 nm) of *P. aeruginosa* following exposure to increasing concentrations of Mānuka honey with UMF grades of (**a**) 5+, (**b**) 10+, (**c**) 12+, (**d**) 15+ and (**e**) 20+, compared with a sugar control. Data represent four independent biological replicates and are presented as mean±sem. Statistical significance relative to the sugar control was determined using multiple *t*-tests with Holm–Sidak correction (*P*<0.05, *P<0.01, **P<0.005 and ***P<0.001).

To determine whether antimicrobial activity could be attributed solely to osmotic stress, bacterial growth responses to Mānuka honey were compared with a sugar control matched to the total sugar composition of honey (Figs 2, 3, 4 and 5).

Across all species tested, Mānuka honey inhibited bacterial growth to a greater extent than the sugar control. For MSSA and MRSA, Mānuka honey rapidly reduced absorbance values as concentration increased, with growth approaching zero at concentrations where the sugar control continued to support measurable proliferation (Figs 2 and 3).

A similar pattern was observed for the Gram-negative species. Although the sugar control produced a gradual concentration-dependent reduction in growth, Mānuka honey consistently caused a more pronounced decline in absorbance (Figs 4 and 5). In *P. aeruginosa*, both treatments produced a slight increase in growth at the lowest concentrations tested before declining with increasing concentration, but inhibition occurred at substantially lower concentrations with honey than with the sugar control.

### Contribution of MGO to the antimicrobial activity of Mānuka honey

To evaluate the contribution of MGO to the antimicrobial activity of Mānuka honey, MGO-matched control solutions were prepared in either distilled water or the sugar control matrix using concentrations equivalent to those quantified in each UMF™ grade (Table 1). MICs were then compared with those obtained for the corresponding Mānuka honey samples.

**Table 1.** MICs (% w/v) of Mānuka honey and MGO-matched control solutions against respiratory pathogens MICs were determined by broth microdilution against MSSA, MRSA, *K. pneumoniae* and *P. aeruginosa*. MGO concentrations for each UMF grade were previously quantified by HPLC. Values represent the lowest concentration resulting in ≥95% inhibition of bacterial growth relative to the untreated control.

		MIC value (%w/v)			
**UMF grade**	**MGO concn (ppm**)	**Species**	**Mānuka honey**	**MGO+water**	**MGO+sugar**
UMF 5+	138	MSSA	20	40	40
		MRSA	15	40	40
		*K. pneumoniae*	30	>40	>40
		*P. aeruginosa*	25	>40	40
UMF 10+	340	MSSA	10	10	10
		MRSA	10	10	10
		*K. pneumoniae*	25	40	40
		*P. aeruginosa*	25	>40	40
UMF 12+	494	MSSA	5	10	10
		MRSA	5	10	10
		*K. pneumoniae*	20	40	40
		*P. aeruginosa*	20	40	40
UMF 15+	623	MSSA	4	10	10
		MRSA	4	10	10
		*K. pneumoniae*	20	40	40
		*P. aeruginosa*	20	40	40
UMF 20+	1,114	MSSA	3	10	10
		MRSA	3	5	10
		*K. pneumoniae*	10	20	20
		*P. aeruginosa*	10	40	40

For the Gram-positive species, MGO-matched solutions only partially reproduced the antimicrobial activity of Mānuka honey. At UMF 5+, Mānuka honey inhibited MSSA and MRSA at 20 and 15% (w/v), respectively, whereas both MGO-matched controls required 40% (w/v). At UMF 10+, MIC values were identical across Mānuka honey, MGO-spiked water and MGO-spiked sugar controls for both MSSA and MRSA. However, this equivalence was not maintained at higher UMF™ grades. From UMF 12+ onwards, Mānuka honey inhibited MSSA and MRSA at substantially lower concentrations than either MGO-matched control, with UMF 20+ honey achieving inhibition at 3% (w/v), compared with 10 and 5–10% (w/v) for the corresponding MGO controls.

For the Gram-negative species, MGO-matched solutions consistently exhibited weaker antimicrobial activity than whole Mānuka honey, with a clear divergence in dose–response patterns. For *P. aeruginosa*, MGO-matched solutions required ≥40% (w/v) across all UMF™ grades, whereas Mānuka honey showed a reduction in MIC from 25% (w/v) at UMF 5+ and 10+ to 20% at UMF 12+ and 15+ and further to 10% at UMF 20+. A similar trend was observed for *K. pneumoniae*, where MGO-matched solutions required >40% (w/v) at UMF 5+ and 40% (w/v) at UMF 10+–15+, with a reduction to 20% (w/v) only at UMF 20+. In contrast, Mānuka honey demonstrated a dose-dependent decrease in MIC from 30% (w/v) at UMF 5+to 10% (w/v) at UMF 20+.

## Discussion

This study demonstrates that Mānuka honey possesses antimicrobial activity against clinically relevant respiratory pathogens and that this activity is influenced by UMF™ grade. All five honey samples inhibited the growth of MSSA, MRSA, *K. pneumoniae* and *P. aeruginosa*, with progressively lower MIC values observed as UMF™ grade increased. The most marked grade-dependent effect was seen for the two *S. aureus* strains, which were inhibited at substantially lower concentrations than the Gram-negative species. These findings are consistent with previous reports showing that Mānuka honey exhibits broad-spectrum antibacterial activity and that Gram-positive bacteria are often more susceptible than Gram-negative organisms [7, 23–25]. They also support observations that higher-grade Mānuka honeys can display greater activity against *S. aureus* [26], although not all studies have reported a straightforward relationship between UMF™ grade and antibacterial potency [7].

The similar susceptibility profiles observed for MSSA and MRSA suggest that methicillin resistance does not materially alter responsiveness to Mānuka honey. This is biologically plausible, as the *mecA*-encoded penicillin-binding protein PBP2a confers resistance specifically to *β*-lactam antibiotics rather than to the multifactorial stresses imposed by honey [27]. In contrast, * K. pneumoniae* and *P. aeruginosa* required substantially higher concentrations for inhibition, in keeping with the greater intrinsic tolerance of Gram-negative bacteria reported previously [7, 28, 29]. The outer membrane of Gram-negative bacteria provides an important permeability barrier, and in species such as *P. aeruginosa*, this is further complemented by efficient efflux systems and marked metabolic adaptability [30, 31]. These intrinsic features may contribute to the higher MICs observed here and may also help explain the modest growth enhancement seen at low concentrations, particularly for *P. aeruginosa*, before inhibition occurred at higher concentrations.

An important control in this study was to assess the extent to which the antimicrobial effects of Mānuka honey could be attributed to its sugar content. High sugar content is recognized to exert antimicrobial effects through osmotic pressure, causing dehydration of cells [13, 14]. In the present study, the sugar control did exert measurable inhibitory effects, confirming its contribution to antibacterial activity. However, across all four species, whole Mānuka honey caused significantly greater inhibition in bacterial growth than the sugar solution. This indicates that osmotic effects alone are insufficient to account for the observed activity. This finding aligns with previous work, showing that the antibacterial potency of Mānuka honey exceeds that of non-Mānuka or sugar-based controls despite broadly comparable sugar composition [15, 16]. However, osmolality was not directly measured in the present study. Although the sugar control was formulated to match the major carbohydrate composition of Mānuka honey, subtle differences in osmotic properties between the honey samples and control solution cannot be excluded. Future studies incorporating direct osmolality measurements would help further define the contribution of osmotic stress to antibacterial activity.

MGO is widely regarded as a defining antimicrobial component of Mānuka honey, and its concentration increases with UMF™ grade [6, 7]. In the present study, MGO-matched control solutions prepared in either water or a sugar solution demonstrated measurable antibacterial activity, confirming that MGO contributes directly to growth inhibition. However, these preparations generally remained less potent than the corresponding whole honey samples. For MSSA and MRSA, MGO controls partially reproduced the activity of the honey, whereas for *K. pneumoniae* and *P. aeruginosa*, they were consistently less active. These data suggest that MGO is an important contributor to antibacterial potency but does not fully explain the activity of whole Mānuka honey. This interpretation is consistent with earlier studies proposing a major role for MGO in antimicrobial action [18, 19], while also supporting reports that the relationship between MGO concentration, UMF™ grade and antibacterial activity is not always linear [7, 20]. Importantly, the inclusion here of both MGO in water and MGO in sugar controls provides a more stringent comparison than approaches considering MGO alone and shows that neither MGO by itself nor MGO combined with a comparable sugar matrix can fully recapitulate the activity of intact honey.

In addition to osmotic effects and MGO, other physicochemical properties and minor constituents of Mānuka honey are likely to contribute, including acidity, phenolic compounds, peptides and additional low-molecular-weight metabolites [6, 32–34]. Previous mechanistic studies also suggest that the downstream effects of Mānuka honey may vary between species. For example, impaired cell division has been described in MRSA following exposure to Mānuka honey [12], whereas in *P. aeruginosa*, changes in membrane-associated proteins and envelope integrity have been reported [35]. The species-dependent susceptibility patterns observed here are therefore likely to reflect both differences in honey composition and differences in bacterial cell envelope structure, physiology and stress responses.

This study has several strengths. It directly compares five UMF™ grades of Mānuka honey against four clinically relevant respiratory pathogens under matched experimental conditions, enabling clear assessment of grade dependence. It also includes both a sugar control and MGO-matched controls, allowing the contribution of two major proposed mechanisms to be examined experimentally rather than inferred. However, translating these findings to clinical use presents important challenges. Although Mānuka honey demonstrated antimicrobial activity *in vitro*, oral consumption would not be expected to achieve therapeutically relevant concentrations at sites of respiratory infection, particularly within the lower airways. Any respiratory application would therefore require formulation-specific development. Potential approaches could include topical delivery to the upper respiratory tract, such as throat sprays, lozenges or gargles, or carefully designed inhaled formulations for lower airway delivery. These approaches would need to address key practical and safety considerations, including viscosity, dose delivery, airway tolerability, sterility, stability and potential effects on the respiratory microbiome. As such, the present findings should be viewed as supporting further mechanistic and formulation-based investigation rather than direct clinical use.

A limitation of this work is that all experiments were performed *in vitro*, and the pathogen panel consisted of single reference strains rather than multiple clinical isolates. In addition, only four respiratory pathogens were examined. Future studies should extend these observations to additional clinically important respiratory species, including *Streptococcus pneumoniae* and *Haemophilus influenzae*, broader collections of clinical isolates and more clinically relevant biofilm and airway infection models. Further work is also required to investigate the mechanisms underlying the antimicrobial activity of Mānuka honey.

## Conclusion

In summary, this study shows that Mānuka honey exerts UMF-dependent antimicrobial activity against clinically relevant respiratory pathogens and that whole Mānuka honey is more inhibitory than either a matched sugar solution or MGO-matched control preparations. These findings indicate that while sugar and MGO both contribute to antibacterial activity, neither (either alone or in combination) is sufficient to account fully for the potency of Mānuka honey. The data support a multifactorial basis for antimicrobial action and provide a foundation for further mechanistic and translational investigation.

## Supplementary material

10.1099/mic.0.001746 Table S1.

## Abbreviations

AMR: antimicrobial resistance
MGO: methylglyoxal
MRSA: methicillin-resistant *Staphylococcus aureus*
MSSA: methicillin-susceptible *Staphylococcus aureus*
UMF: Unique Mānuka Factor
