# Supplementary material for: Unique Mānuka Factor (UMF)-dependent antimicrobial activity of Mānuka honey against respiratory pathogens cannot be explained by sugar and methylglyoxal alone
Source: Microbiology (Reading). 2026 Aug 11;172(8):001746. doi: 10.1099/mic.0.001746 (PMC13460517; doi:10.1099/mic.0.001746)
Supplement: Supplementary file 1 [file mic-172-01746-s001.pdf]

Supplementary Table S1. Batch-specific compositional characteristics of the Mānuka honey samples used in this study.

Data were provided by Comvita (New Zealand) and include authenticity markers routinely measured as part of UMF™ certification and Mānuka honey characterisation. DHA, dihydroxyacetone; MGO, methylglyoxal; HMF, hydroxymethylfurfural; 3-PLA, 3-phenyllactic acid; 4-HPLA, 4-hydroxyphenyllactic acid.

Mānuka honey type	Batch number	DHA (ppm)	MGO (ppm)	HMF (ppm)	Leptosperin (ppm)	3-PLA (ppm)	4-HPLA (ppm)
UMF 5+	34374901	226	138	17.3	195	502	3.9
UMF 10+	34327090	733	340	22.6	362	707	5.9
UMF 12+	34363853	795	494	30.5	391	868	7.8
UMF 15+	34213919	1078	623	24.1	459	1085	8.1
UMF 20+	34327121	1770	1114	33.2	1160	1141	9.8
